# Anxiety and Depression After a Cardiac Event: Prevalence and Predictors

**DOI:** 10.3389/fpsyg.2019.03010

**Published:** 2020-01-29

**Authors:** Barbara Murphy, Michael Le Grande, Marlies Alvarenga, Marian Worcester, Alun Jackson

**Affiliations:** ^1^Australian Centre for Heart Health, Melbourne, VIC, Australia; ^2^Faculty of Health, Deakin University, Burwood, VIC, Australia; ^3^Department of Psychology, The University of Melbourne, Parkville, VIC, Australia; ^4^Faculty of Health, Federation University Australia, Ballarat, VIC, Australia; ^5^Department of Epidemiology and Preventive Medicine, Monash University, Melbourne, VIC, Australia; ^6^Centre on Behavioral Health, The University of Hong Kong, Hong Kong, Hong Kong

**Keywords:** psychosocial risk factors, heart disease, anxiety, depression, heart attack

## Abstract

**Introduction:**

Patients who are anxious or depressed after an acute cardiac event are at increased risk of a subsequent event and premature death. It is therefore important to identify these patients early in order to initiate supportive or even preventive measures. In the present study, we report on the prevalence of anxiety and depression during the first 12 months after an acute cardiac event, and the patient characteristics predictive of increased anxiety and depression risk in early and late convalescence.

**Methods:**

We recruited a sample of 911 patients with acute myocardial infarction (AMI), acute coronary syndrome (ACS), and/or unstable angina (UA), and/or undergoing coronary artery bypass graft surgery (CABGS). Patients completed the Hospital Anxiety and Depression Scale (HADS) close to the time of their event, and again during early (2–4 months post-event) and late (6–12 months post-event) convalescence. Using HADS-A and HADS-D cut-offs of 8+, prevalence rates for anxiety, depression, and comorbid anxiety and depression were determined for each timepoint. Chi-square tests and odds ratios were used to identify baseline patient characteristics associated with increased anxiety and depression risk over 12 months.

**Results:**

Anxiety rates were 43, 28, and 27% at the time of the event, early, and late convalescence. Depression rates were 22, 17, and 15%, respectively. Factors consistently associated with increased anxiety and depression risk were history of depression, financial strain, poor self-rated health, low socioeconomic status, younger age (<55 years), and smoking. Obesity, diabetes, and social isolation (living alone or being unpartnered) were identified as important albeit less significant risk factors. Neither sex nor event type were predictive of anxiety or depression.

**Conclusion:**

This large patient sample provided the opportunity to identify rates of anxiety and depression during the 12 months after a cardiac event and key patient characteristics for increased risk. These risk factors are easily identifiable at the time of the event, and could be used to guide the targeting of support programs for patients at risk.

## Introduction

Anxiety and depression are both common after an acute cardiac event, such as acute myocardial infarction (AMI) or coronary artery bypass graft surgery (CABGS). It is generally agreed that around one in five patients meet diagnostic criteria for depression while hospitalized for a cardiac event (Thombs et al., 2006; Lichtman et al., 2008; Colquhoun et al., 2013; Murphy et al., 2016), and up to one in three experience severe anxiety (Andrew et al., 2000; Tully and Baker, 2012; Murphy et al., 2016). While relatively few studies have reported rates of anxiety and depression at later points during patients’ convalescence, there is evidence that early symptoms resolve for many patients during the first few months after hospital discharge (Murphy et al., 2008a, 2013, 2016).

Patients who are anxious or depressed after an acute cardiac event are at increased risk of a subsequent event and premature death (Strik et al., 2003; Barth et al., 2004; van Melle et al., 2004; Tully et al., 2013). The negative health impacts appear to surface or increase when anxiety or depressive symptoms persist or emerge after hospital discharge during convalescence (Blumenthal et al., 2003; Murphy et al., 2013; Worcester et al., 2019; Kim et al., 2020). In a 12-year follow-up study of 170 female AMI and CABGS patients, we found that the mortality rate was highest in those whose depression symptoms worsened in the 2 months after hospital discharge, and lowest in those whose in-hospital symptoms remitted by 2 months (Murphy et al., 2013). Others have reported similar findings (Blumenthal et al., 2003). Anxiety at one to 2 months post-event has been found to confer a 2.3 to 2.8-fold increased risk of adverse cardiac events (Strik et al., 2003; Frasure-Smith and Lesperance, 2008). For this reason, it is important to identify patients at risk of symptoms of anxiety or depression that emerge later or persist into convalescence, rather than identifying only those with symptoms present in hospital (Murphy et al., 2016). Moreover, it is important to identify patient characteristics other than early anxious or depressive symptoms, given that these cannot be regarded as a good indicator of later mental health status (Murphy et al., 2008a, 2013, 2016).

Mechanisms for the associations between CHD and mental health have been well documented in several reviews published in recent years. These include hypothalamic pituitary adrenal axis dysregulation, platelet activation, and inflammation (Brydon et al., 2006; Smith and Blumenthal, 2011; Goldstein et al., 2015). It has been suggested that CHD and anxiety and depression share several biological mechanisms. Patients with depression, for example, have higher levels of biomarkers that promote atherosclerosis; in anxiety and depression, we see reduced heart rate variability suggesting decreased parasympathetic activity; altered serotonergic pathways; altered platelet aggregability; and increased C-reactive protein, an indicator of increased inflammatory response. The process can be summarized in the following way (Brydon et al., 2006): atherosclerosis, the disorder underlying this disease, is an inflammatory process in which leukocytes interact with structurally intact but dysfunctional endothelium of the arteries. Platelets bind to leukocytes and promote their recruitment to the endothelium. Platelet-leukocyte interactions also stimulate the release of pro-inflammatory and pro-thrombotic factors which promote atherosclerosis (Brydon et al., 2006). Another mechanism involves impaired cardiac neuronal reuptake of noradrenaline across the heart in patients with anxiety and depression (Alvarenga et al., 2006); such an abnormality magnifies sympathetically mediated responses, particularly emotionally driven responses in the heart where noradrenaline inactivation is so dependent on neuronal reuptake, potentially causing sensitization to cardiac symptom development. Augmentation of the sympathetic neural signal in the heart, by impairment of neuronal noradrenaline reuptake, additionally could increase cardiac risk, predisposing to the development of cardiac tachyarrhythmia (Alvarenga et al., 2006).

Several effective evidence-based supports are available to assist patients to manage anxiety and depression, underscoring the importance of early identification of at-risk patients. Cognitive behaviour therapy (CBT) has been shown to be effective in reducing anxiety and depression in cardiac patients (Ski et al., 2016; Tully et al., 2017), as has cost-effective short term psychotherapy (Pristipino et al., 2019) and low intensity collaborative care (Huffman et al., 2014). Selective serotonin reuptake inhibitors (SSRIs) are effective for cardiac patients with depression, both for remission of depressive symptoms (Thombs et al., 2008; Pizzi et al., 2011; Kim et al., 2018) and reduced incidents of re-events and death (Taylor et al., 2005; Kim et al., 2018). Physical activity has been shown to be effective in reducing symptoms of mild to moderate depression in patients generally (Wegner et al., 2014), and in older patients with chronic disease (Blumenthal et al., 1999).

If patients at risk of anxiety and/or depression during convalescence after an acute event are identified at the time of their event, supportive or even preventive measures can be initiated. Therefore, it is important to identify baseline characteristics – or risk factors – for anxiety and depression during convalescence.

### Aims of the Study

The present study has two primary aims. First, the study aims to report the prevalence of anxiety and depression at the time of the acute cardiac event, and again at early and late convalescence during the first year after hospital discharge. Second, the study aims to identify the patient characteristics associated with increased risk of anxiety and depression during early and late convalescence.

## Materials and Methods

### Sample

Eligible patients were those admitted to hospital after AMI, acute coronary syndrome (ACS) or unstable angina (UA), and those on the waiting list for elective CABGS. Admissions were to four hospitals in metropolitan Melbourne, Victoria, and two hospitals in regional Victoria, Australia. Patients with a current episode of severe psychiatric illness, those with intellectual disability, and those who did not have sufficient English language to complete the questionnaires were excluded.

### Procedure

Eligible patients were identified by medical or nursing staff from relevant admission records and waiting lists and were then approached by the researcher who explained the study and provided an introductory letter and consent form. All participating patients provided signed consent. All patients completed a baseline self-report questionnaire (while in hospital after admission for AMI, ACS, and UA, and prior to hospital admission for elective CABGS patients), and again during early convalescence (2–4 months post-event) and late convalescence (6–12 months post-event). Baseline questionnaires were completed and collected by the researcher, while follow-up questionnaires to re-assess anxiety and depression were mailed to patients’ homes, with completed questionnaires being returned by patients in reply paid envelopes. Approval was obtained from the Human Research Ethics Committees of the participating hospitals.

### Measures

The 14-item *Hospital Anxiety and Depression Scale* (HADS) was used to assess anxiety (HADS-A) and depression (HADS-D) at each of the three timepoints (Snaith and Zigmond, 1994). The HADS has been shown to be psychometrically sound for use in cardiac populations (Roberts et al., 2001). The generic cut offs for mild to moderate symptoms are HADS-A > 8 for anxiety and HADS-D > 8 for depression. It was necessary to dichotomize HADS scores in this way in order to report on prevalence rates for anxiety and depression, as per the first aim of the study.

*Socio-demographic information* was collected at baseline by self-report questionnaire, and included age, sex (male vs. female), partner status (partnered vs. unpartnered), living arrangements (alone vs. with other), current or last occupation (non-manual vs. manual), and private health cover (insured vs. uninsured). Age was dichotomized as under 55 vs. 55 or over. In Australia, having private health cover is associated with outright home ownership, luxury vehicle ownership and a six-figure income (Muggli et al., 2006), hence its relevance as an indicator of financial security. Manual occupation and not having private health cover were combined to create a variable for socioeconomic status (SES), with patients classified as manual workers and having no health cover being classified as low SES. *Financial strain* was assessed by asking patients to rate their level of financial strain, as either extreme, considerable, moderate, slight or none. For the dichotomous variable, ratings of extreme and considerable were combined to indicate financial strain.

*Clinical information* was also collected at baseline by self-report questionnaire, and included admission event (AMI, CABGS, ACS, and UA), diabetes mellitus, height, and weight (for calculating body mass index; dichotomized as obese vs. not obese) and smoking status (current, past or never-smoked, dichotomized as current vs. non-smoker). While there is inevitable overlap between the diagnostic categories of AMI, CABGS, ACS, and UA, patients’ diagnoses were allocated based on their index event at the time of hospital admission. *Self-rated health* was assessed on a 5-point scale, indicated as poor, fair, good, very good, and excellent (Bosworth et al., 1999). Responses were re-classified as poor vs. not poor for the dichotomous variable. *History of depression* was assessed by asking patients “Have you ever been depressed in the past, prior to your cardiac event?” and were classified as yes or no.

### Data Analysis

The prevalence of anxiety and depression was calculated for each of the three timepoints: at the time of the event, early convalescence and late convalescence. The chi-square statistic, crude odds ratios and 95% confidence intervals were used to identify baseline factors significantly associated with increased risk of anxiety, depression and comorbid anxiety/depression at either early or late convalescence. We identified the variables that might be clinically significant and show an association or borderline association with clinical anxiety or depression, with variables *p* < 0.10 reserved for inclusion in the multivariate analysis. We included comorbid anxiety/depression as an outcome in this level of the analysis given its clinical importance as a predictor of poorer outcomes than either disorder alone (Coplan et al., 2015).

We used decision-tree analysis using Chi-square automatic interaction detection (CHAID) (Kass, 1980) to determine clinically relevant predictors of anxiety or depression at late convalescence. We chose anxiety OR depression as the outcome at this level of the analysis in order to capture the largest number of patients with any mental health condition and therefore maximize the statistical power of the analysis. Moreover, from a clinical perspective, it is useful to identify predictors for *either* mental health condition in order to best identify at-risk patients, be they those at risk of anxiety or those at risk of depression. We chose to present the CHAID analysis only for predicting anxiety or depression at late convalescence since this is a period when most of the adverse effects of surgery and treatment have stabilized and any ongoing elevated anxiety and depression is likely to be of clinical significance (Milani et al., 1996; Worcester et al., 2007). We chose the CHAID method since it is intended to work with discrete targets and does not require the data to be normally distributed (Song and Lu, 2015). The CHAID algorithm also has the advantage of including missing values as part of the analysis. Based on the smallest *p* value, the algorithm decides whether to merge the missing category with its most similar category or to keep the missing category as a separate category. The classification tree starts with identifying the target variable (anxiety or depression) which would be considered the root. CHAID analysis then splits the target into two or more categories (initial parent nodes). Using Bonferroni correction as a splitting criterion, these nodes are then split into child nodes. In this CHAID analysis tree, the variable that is a major influence on anxiety or depression appears first in the tree (initial node) and the less influential variables come last (terminal nodes). In order to preserve statistical power, the number of splits was limited to three and number of patients in child nodes was restricted to 20 and above, thereby restricting the number of levels. Similar criteria have been used in previous decision tree analyses involving similar size samples (Reges et al., 2014). To validate resultant decision-trees a 5-fold cross-validation procedure was applied with misclassification risk estimate, overall accuracy percentage and cross-validation risk estimate calculated.

All analyses were conducted using IBM SPSS Statistics V.26 (IBM Corporation, Armonk, NY, United States).

## Results

### Characteristics of the Sample

**FIGURE 1:**
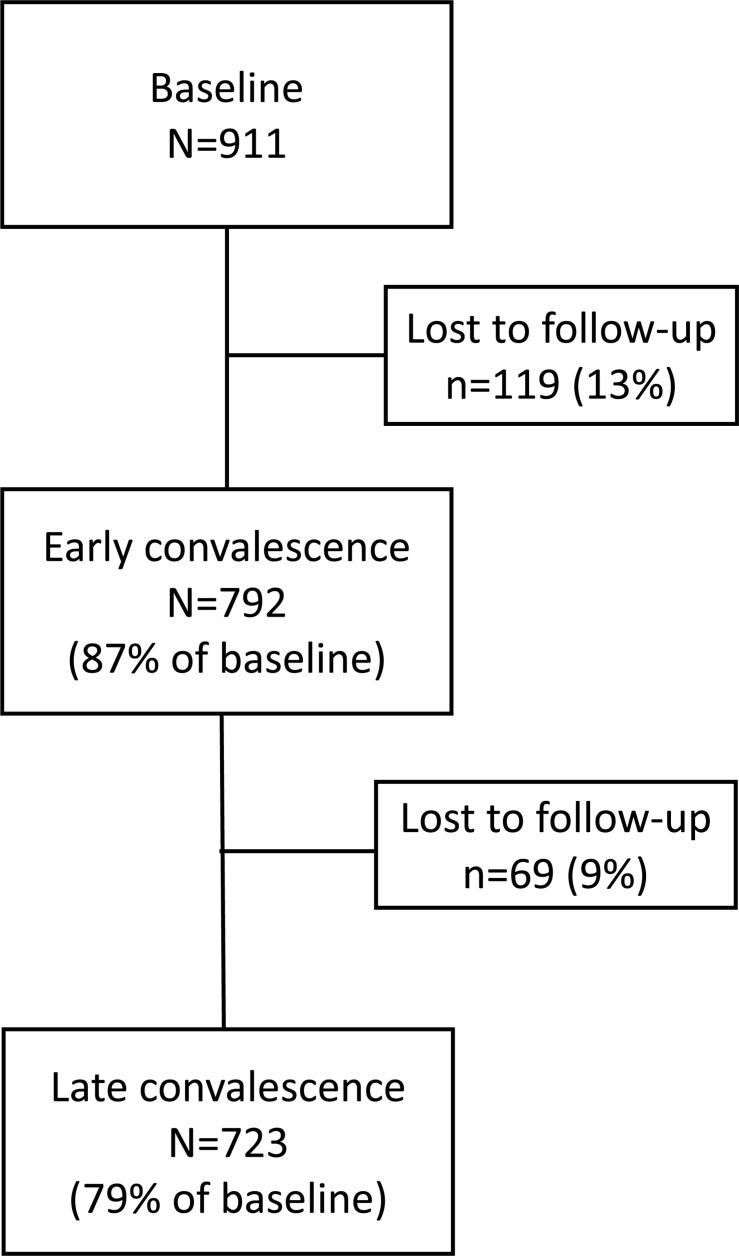
Flow of patients through the study from baseline to late convalescence.

In total, 911 patients were recruited at baseline, 792 (87%) completed the first follow-up assessment during early convalescence (2–4 months post-event) and 723 (79%) completed the second follow-up assessment during late convalescence (6–12 months post-event). The flow of patients through the study is shown in Figure 1.

Sociodemographic and clinical information for the sample of 911 patients is shown in Table 1. As shown, two thirds of the patients were male, and almost three quarters were over 55 years of age. Almost four out of five lived with other(s), and around two thirds were partnered. Over half were or had been in non-manual occupations. While almost two thirds had no health insurance, fewer than one in four were classified as being of low SES and just under 10% were experiencing extreme or considerable financial strain. Most patients were admitted following AMI or to undergo CABGS. Almost one in five were smokers at the time of admission, almost one in four had diabetes, one in three was obese, and over one in four had a history of depression. Just over one in ten rated their health as poor.

**TABLE 1:** Characteristics of the sample.

		***N***	***n***	**%**
**Sociodemographic characteristics**				
Sex	Male	911	605	66.4
	Female		306	33.6
Aged under 55		911	247	27.1
Lives alone		909	195	21.5
Unpartnered		896	291	32.5
Manual occupation		711	287	40.4
Low socioeconomic status		635	149	23.5
No private health insurance		552	334	60.5
Extreme or considerable financial strain		547	49	9.0
**Clinical characteristics**				
Event type	Coronary artery bypass graft surgery	911	491	35.2
	Acute myocardial infarction		317	54.5
	Acute coronary syndrome/unstable angina		93	10.3
Current smoker		910	172	18.9
Diabetes mellitus		900	206	22.9
Poor self-rated health		835	97	11.6
Obese		639	205	32.1
History of depression		491	140	28.5
**Anxiety and depression rates**				
Anxious or depressed	Baseline	910	440	48.4
	Early convalescence	791	257	32.5
	Late convalescence	723	222	30.7
Anxious	Baseline	911	394	43.2
	Early convalescence	792	225	28.4
	Late convalescence	723	197	27.2
Depressed	Baseline	910	205	22.5
	Early convalescence	791	136	17.2
	Late convalescence	723	107	14.8
Anxious and depressed	Baseline	911	159	17.5
	Early convalescence	792	104	13.1
	Late convalescence	723	82	11.3

**N* = 911 at baseline; missing data on some variables due to variations in baseline questionnaires used at different hospitals. Low socioeconomic status based on being in manual occupation and having no private health insurance. Anxious = HADS-A ≥ 8; Depression = HADS-D ≥ 8; Early convalescence = 2–4 months post-event; Late convalescence = 6–12 months post-event.*

### Prevalence of Anxiety and Depression at Baseline, Early, and Late Convalescence

The number and proportion of patients classified as anxious and/or depressed at each of the three timepoints are also shown in Table 1. More than two in five patients were anxious at the time of the event and more than one in four were anxious during early and late convalescence. Rates were slightly lower for depression: over one in five patients were depressed at the time of their event, with under one in five depressed during early and late convalescence. When totaled, almost half the patients were *either* anxious or depressed at the time of the event, with rates of 32% for early and 31% for late convalescence.

In terms of comorbidity, almost one in five patients were *both* anxious and depressed at the time of the event, with rates of 13 and 11% for early and late convalescence, respectively. Indeed, there was a significant overlap between anxiety and depression at both early (χ2 = 186.11, df = 1, *p* < 0.001) and late (χ2 = 154.53, df = 1, *p* < 0.001) convalescence. At early convalescence, 46% of patients who were anxious were also depressed, while 76% of those who were depressed were also anxious. At late convalescence, these overlap rates were 42 and 77% respectively.

### Predictors of Anxiety in Early and Late Convalescence

The factors significantly associated with anxiety in early and late convalescence are shown in Table 2. For each factor, the table shows the proportion of patients classified as anxious, together with the odds ratios, 95% confidence intervals, chi-square value and *p*-value.

**TABLE 2:** Factors associated with *anxiety* in early and late convalescence.

	***N***	**Proportion anxious (%)**	**Odds ratio Exp(B)**	**95% CIs**	**Chi-square**	**df**	***p***
**Early convalescence**							
Overall	792	28					
Depression history	434	45	2.96	1.89–4.64	23.43	1	<0.001
Financial strain	486	56	2.77	1.39–5.50	9.02	1	0.003
Aged under 55	792	40	2.05	1.45–2.88	17.04	1	<0.001
Poor self-rated health	691	42	2.00	1.25–3.22	8.46	1	0.003
Low SES	522	36	1.68	1.09–2.60	5.53	1	0.019
Smoker	790	37	1.62	1.10–2.37	6.19	1	0.013
Obese	565	30	1.54	1.03–2.29	4.45	1	0.035
Unpartnered	778	32	1.35	0.98–1.87	3.30	1	0.069
**Late convalescence**							
Overall	723	27					
Financial strain	440	71	5.94	2.55–13.86	20.98	1	<0.001
Depression history	415	48	3.54	2.24–5.59	31.07	1	<0.001
Aged under 55	723	43	2.60	1.80–3.76	26.84	1	<0.001
Poor self-rated health	629	44	2.35	1.41–3.93	11.13	1	0.001
Smoker	721	40	2.00	1.34–3.00	11.75	1	0.001
Low SES	480	38	1.84	1.16–2.93	6.85	1	0.009
Obese	524	30	1.62	1.06–2.47	4.96	1	0.026

*Anxious = HADS-A ≥ 8; Early convalescence = 2–4 months post-event; Late convalescence = 6–12 months post-event. CI, confidence intervals; SES, socioeconomic status. Statistical test, chi-square. Significance level set at *p* < 0.1. All predictors are presented in descending order in terms of odds ratios. For early convalescence associations with living alone, diabetes, sex, and event type were *p* ≥ 0.1. For late convalescence associations with living alone, partner status, diabetes, sex and event type were *p* ≥ 0.1.*

As shown in Table 2, the rate of anxiety in early and late convalescence was significantly increased for patients with a history of depression, those aged under 55, those reporting moderate or severe financial strain, those with poor self-rated health, smokers, those classified as low SES, and those classified as obese. Having a depression history conferred a 3 to 3.5-fold increased risk of early and late anxiety, while financial strain conferred an almost 3-fold increased risk of early anxiety and an almost 6-fold increased risk of late anxiety. Being aged under 55 and having poor self-rated health each conferred a 2-fold or greater increased risk of early and late anxiety. Smoking conferred a 60% increased risk of early anxiety and doubled the risk of later anxiety. Low SES and obesity each conferred at least a 30% increased risk of anxiety in early and late convalescence. Being unpartnered also marginally increased the risk of early anxiety. Sex, event type, living arrangements, and diabetes were not predictive of anxiety at either timepoint during convalescence.

### Predictors of Depression in Early and Late Convalescence

The factors significantly associated with depression in early and late convalescence are shown in Table 3. Financial strain conferred a 4 to 5-fold increased risk of early and late depression, while poor self-rated health conferred over a 3-fold increased risk. Having a history of depression conferred a 2.5-fold and 3.4-fold increased risk of early and late depression, respectively. Low SES, age under 55 and smoking each conferred around a 2-fold increased risk at both timepoints. Being unpartnered and living alone both significantly increased the risk of late but not early depression. Obesity and diabetes increased the risk of early or late depression, respectively, although their effects were less statistically significant than other factors. Neither sex nor event type were predictive of depression at either timepoint.

**TABLE 3:** Factors associated with *depression* in early and late convalescence.

	***N***	**Proportion depressed (%)**	**Odds ratio Exp(B)**	**95% CIs**	**Chi-square**	**df**	***p***
**Early convalescence**							
Overall	791	17					
Financial strain	485	42	3.93	1.93–8.00	16.09	1	<0.001
Poor self-rated health	690	35	3.37	2.03–5.59	23.96	1	<0.001
Depression history	434	26	2.57	1.51–4.37	12.64	1	<0.001
Low SES	522	26	2.21	1.34–3.65	10.07	1	0.002
Aged under 55	791	24	1.78	1.19–2.66	8.15	1	0.004
Smoker	789	24	1.75	1.13–2.71	6.43	1	0.011
Obese	564	19	1.57	0.97–2.55	3.39	1	0.066
Unpartnered	777	20	1.38	0.94–2.04	2.75	1	0.097
**Late convalescence**							
Overall	723	15					
Financial strain	440	39	4.91	2.17–11.09	17.25	1	<0.001
Depression history	415	26	3.42	1.95–6.03	19.64	1	<0.001
Poor self-rated health	629	29	2.81	1.58–5.00	13.20	1	<0.001
Aged under 55	723	24	2.30	1.48–3.57	14.23	1	<0.001
Low SES	480	24	2.11	1.22–3.66	7.39	1	0.007
Smoker	721	22	1.87	1.15–3.02	6.58	1	0.010
Unpartnered	711	20	1.71	1.10–2.68	6.26	1	0.012
Lives alone	720	20	1.65	1.04–2.61	4.66	1	0.031
Diabetes	718	19	1.50	0.95–2.38	3.03	1	0.082

*Depressed = HADS-D ≥ 8; Early convalescence = 2–4 months post-event; Late convalescence = 6–12 months post-event; CI, confidence intervals; SES, socioeconomic status. Statistical test, chi-square. Significance level set at *p* < 0.1. All predictors are presented in descending order in terms of odds ratios. For early convalescence associations with living alone, diabetes, sex and event type were *p* ≥ 0.1. For late convalescence associations with obesity, sex, and event type were *p* ≥ 0.1.*

### Predictors of Comorbid Anxiety and Depression in Early and Convalescence

The factors significantly associated with being both anxious and depressed in early and late convalescence are shown in Table 4. For both early and late comorbid anxiety and depression, there were again six common risk factors: financial strain, history of depression, poor self-rated health, being aged under 55, low SES and smoking. Financial strain conferred a 3.8 increased risk of comorbid anxiety and depression in early convalescence and an almost 5-fold risk in later convalescence. Depression history conferred an almost 4-fold increased risk at both timepoints, while poor self-rated health carried around a 3-fold increased risk. Being under 55 and smoking each carried an increased risk of around 2–2.5. Being unpartnered, living alone, being obese and having diabetes each contributed to the risk of comorbid anxiety and depression at some point during the convalescent period, although the strength of these factors was relatively less statistically significant. Neither sex nor event type were predictive of comorbid anxiety and depression at either timepoint during convalescence.

**TABLE 4:** Factors associated with *comorbid anxiety and depression* in early and late convalescence.

	***N***	**Proportion comorbid A and D (%)**	**Odds ratio Exp(B)**	**95% CIs**	**Chi-square**	**df**	***p***
**Early convalescence**							
Overall	792	13					
Financial strain	486	36	3.82	1.83–7.96	14.41	1	<0.001
Depression history	434	25	3.50	1.96–6.09	19.87	1	<0.001
Poor self-rated health	691	27	2.97	1.71–5.14	16.13	1	<0.001
Low SES	522	22	2.52	1.47–4.31	11.99	1	0.001
Aged under 55	792	21	2.20	1.43–3.40	13.18	1	<0.001
Smoker	790	20	1.95	1.21–3.13	7.83	1	0.005
Obese	565	14	1.72	0.99–2.99	3.76	1	0.052
Unpartnered	778	16	1.44	0.94–2.21	2.80	1	0.095
**Late convalescence**							
Overall	723	11					
Financial strain	440	36	4.90	2.12–11.30	16.39	1	<0.001
Depression history	415	23	3.71	2.01–6.83	19.42	1	<0.001
Poor self-rated health	629	23	2.95	1.57–5.52	12.31	1	<0.001
Aged under 55	723	20	2.52	1.55–4.08	14.69	1	<0.001
Low SES	480	21	2.46	1.37–4.44	9.40	1	0.002
Smoker	721	19	2.18	1.29–3.67	8.92	1	0.003
Unpartnered	711	15	1.76	1.10–2.83	5.67	1	0.017
Lives alone	720	16	1.68	1.01–2.80	4.10	1	0.043
Diabetes mellitus	718	15	1.48	0.90–2.47	2.28	1	0.088

*Comorbid anxiety and depression = HADS-A ≥ 8 and HADS-D ≥ 8; Early convalescence = 2–4 months post-event; Late convalescence = 6–12 months post-event; CI, confidence intervals; SES, socioeconomic status. Statistical test, chi-square. Significance level set at *p* < 0.1. All predictors are presented in descending order in terms of odds ratios. For early convalescence associations with living alone, diabetes, sex, and event type were *p* ≥ 0.1. For late convalescence associations with obesity, sex, and event type were *p* ≥ 0.1.*

### Cumulative Impact of Predictors of Anxiety and Depression

**FIGURE 2:**
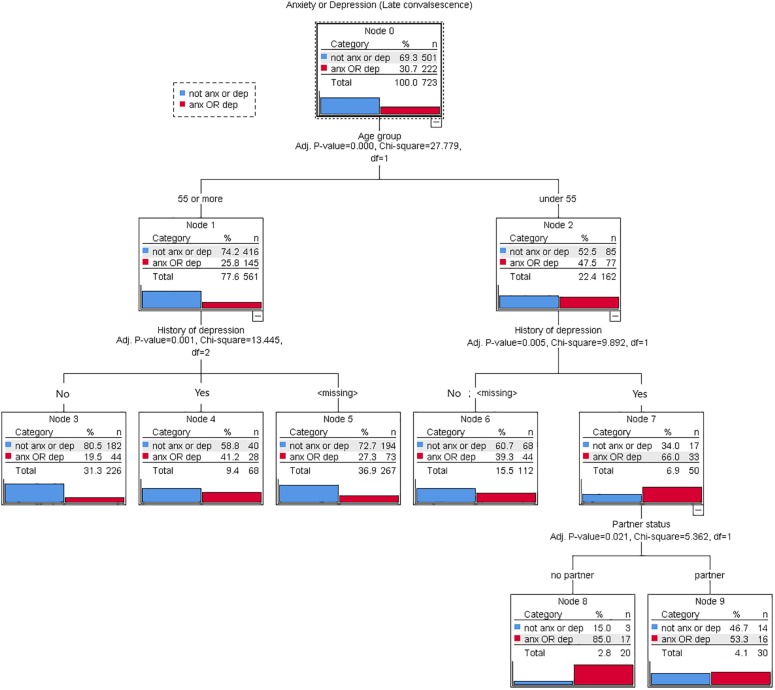
Decision tree (using the CHAID algorithm) for predicting presence of anxiety or depression in late convalescence among 723 patients admitted to hospital for acute myocardial infarction (AMI), coronary artery bypass graft surgery (CABGS), acute coronary syndrome (ACS), percutaneous coronary syndrome (PCI), or angina. At each node the best predictor for anxiety or depression was selected from the six key potential predictors identified in bivariate analyses, and the optimal forecasting values were determined.

**FIGURE 3:**
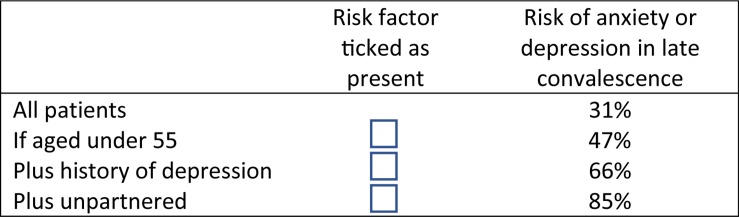
Cumulative risk of anxiety or depression for three key risk factors.

Using decision tree analysis, the cumulative impact of multiple risk factors was demonstrated. The single most critical factor (initial node) determining presence of *either* anxiety or depression in late convalescence was age: 47% of patients under 55 were anxious or depressed, compared with only 26% of patients aged 55 or more. Amongst those aged under 55, the most important contributor to anxiety/depression risk was history of depression: 66% of those with a depression history were anxious or depressed in late convalescence, compared to only 39% with no depression history or an unknown history. The next most important contributor was partner status: amongst those aged under 55, with a depression history and no partner, 85% were anxious or depressed, compared to 53% of their partnered counterparts. Amongst those age 55 or more, again history of depression was the most important contributor to risk: 41% of those with a depression history were anxious or depressed in late convalescence, compared with only 19% of those without a history. The classification tree analysis accurately predicted patients’ anxiety or depression status (71.5% correct). The cross-validation estimate for risk of misclassification (0.30) approximated that of the full model (0.28). Findings of the decision tree analysis are shown in Figures 2, 3.

## Discussion

In this large sample of cardiac patients, the rates of anxiety were 28 and 27% in early and late convalescence, while the rates of depression were 17 and 15% respectively. Not surprisingly, these rates are substantially higher than seen in the Australian population where 12-month prevalence rates are 14% for anxiety disorders and 6% for depressive disorders (Australian Bureau of Statistics, 2008). These figures suggest that in the 12 months after an acute event, the prevalence rates increase 2-fold for anxiety and 2.5-fold for depression. Moreover, these rates of anxiety and depression are largely consistent with those seen after other traumatic events such as stroke (Broomfield et al., 2014) and cancer diagnosis (Linden et al., 2012).

Notably 13% of patients had *comorbid* anxiety and depression in early convalescence and 11% in late convalescence. This is significant as the overlap of anxiety and depression complicates both diagnosis and treatment (Coplan et al., 2015). Comorbid anxiety with depression predicts poorer outcomes than either disorder occurring alone, with a higher rate of treatment resistance (Coplan et al., 2015). The complexity of treatment underscores the importance of identifying patients at risk of mental health comorbidity in the year after a cardiac event.

At the time of the event, almost half the patients reported *either* anxiety or depression. However, symptoms resolved for many patients, with most resolution occurring during early convalescence from hospital discharge to the 2 to 4-month mark. This is consistent with other studies that have demonstrated a resolution of symptoms for many patients during the early post-event period (Murphy et al., 2008a, 2013, 2016). Nonetheless, almost a third of patients had symptoms at the 6 to 12-month mark.

The present study has successfully identified some key risk factors for anxiety and depression in early and late post-event convalescence. Factors consistently associated with increased risk included having been depressed prior to the event, experiencing financial strain, rating one’s health poorly, being of low SES, being younger, and smoking. A patient’s mental health history has previously been identified as a key risk factor for anxiety and depression (Martens et al., 2008), as have younger age (Cheok et al., 2003), low SES and financial difficulties (Cheok et al., 2003; Freeman et al., 2016), poorer health status (Cheok et al., 2003), and smoking (Perez et al., 2008; Tjora et al., 2014; Sever et al., 2019). One recent study identified financial strain as a predictor of recurrent cardiac events in CHD patients, highlighting its negative prognostic impact (Georgiades et al., 2009). Another linked financial hardship to job loss, also highlighting its negative impacts on both physical and mental health outcomes (Warraich et al., 2018).

Indeed, financial strain was identified as an important and strong predictor, and screening for it could easily be implemented in clinical practice. The present study used a simple 5-point scale (from not at all to extreme financial strain) and demonstrated that those self-reporting as experiencing either considerable or extreme financial strain were at substantially increased risk of poor mental health outcomes post cardiac event. Indeed, screening for all the factors identified here would serve as a useful approach to identifying patients at risk. All the factors included in the present study could be easily assessed in hospital or at discharge.

There was also evidence of increased risk for socially isolated patients, indicated by significant associations between both living alone and being unpartnered with depression at late convalescence, as well as through the decision tree analysis. Again this is consistent with earlier research that has demonstrated that social isolation is associated with poor mental health including depression and suicide (Cheok et al., 2003; Iliffe et al., 2007; Manemann et al., 2018). Similarly, lack of marital support has been shown to significant predict adverse outcomes (Compare et al., 2013). Social isolation has also been shown to be an important contributor to hospital readmission in both heart disease (Murphy et al., 2008b) and heart failure patients (Saito et al., 2018). Moreover, the general health risk associated with social isolation is comparable to that associated with smoking (Iliffe et al., 2007). Notably, it is possible that the decision tree analysis was more effective in identifying partner status as a predictor than was the bivariate analyses, given its multivariate nature and ability to identify predictors of unique variance.

Obesity and diabetes were also identified as important albeit less significant risk factors. Again, both these factors have been identified previously as risk factors for depression in particular (Frasure-Smith et al., 2000). For people with diabetes, having a comorbid condition such as CHD can have a profound effect, complicating the treatment regime, undermining medication adherence and compromising diabetes self-care (Piette and Kerr, 2006). When comorbid illnesses need to be comanaged, vulnerability to mental health problems can increase. Likewise, the relationship between obesity and depression is well established (Luppino et al., 2010). Indeed, meta-analyses have confirmed a reciprocal relationship between the two conditions, whereby obesity increases the risk of depression and, conversely, depression is predictive of developing obesity (Luppino et al., 2010).

The results of the decision tree analysis highlight the cumulative effect of two or more risk factors in increasing risks of post-event anxiety and depression, demonstrating that having multiple risk factors can substantially elevate one’s mental health risk. Being under 55 was identified as the single most important risk factor for *either* anxiety or depression in late convalescence, with history of depression and being unpartnered further contributing to the risk for these younger patients. Almost all (85%) patients with all three risk factors – aged under 55, having a depression history and being unpartnered – were either anxious or depressed in late convalescence. Figure 3 shows how the factors could be combined and used as a simple screening tool in clinical practice.

It is notable that neither sex nor event type were predictive of anxiety or depression at any timepoint. Some studies have reported higher rates of post-event depression in women (Cheok et al., 2003), possibly due to higher rates of help-seeking and reporting amongst women compared to men. On the other hand, other studies report higher depression in men (Sever et al., 2019). Further, there is some evidence that AMI patients experience more anxiety and depression than CABGS patients in early convalescence (Westin et al., 1997), although there is no evidence that this difference is sustained (Westin et al., 1997; Moser et al., 2010). Importantly, the possibility of overlap between the diagnostic categories, the degree of which was not recorded in the present study, might in part explain the null findings for this variable.

### Limitations

There are some study limitations that should be noted. First, there was substantial attrition of participants over time, from 911 at baseline to 723 (79%) by the late convalescent follow-up. This is typical of longitudinal studies of this type, particularly where mailed questionnaires are used. Efforts were made to contact patients and next of kin to optimize participation of survivors over the study period. Second, there was also considerable missing data for several independent variables, most notably history of depression, financial strain, SES, and obesity. Unfortunately, these data were not collected from all participants, with different baseline questionnaires being used at the different participating hospitals. Nonetheless, the sample for each of these variables is still relatively large and therefore the results can be regarded as valid. Moreover, the missing data was not due to skipped items or other systematic bias. Third, we did not record patients’ treatment, if any, for anxiety or depression, including the use of anti-depressive or anti-anxiolytic medication, CBT or other treatments. We were therefore unable to control for these factors in the analysis. Similarly, we did not systematically collect data on patients’ illness severity, although their self-reported health rating provides a good indication of this and, not surprisingly, strongly predicted both anxiety and depression. Finally, we did not collect information on patients’ dietary habits or physical activity levels, both of which are important factors in mental health and wellbeing (Sever et al., 2019).

Two of the key predictors – patients’ history of depression and financial strain – were established using subjective self-report measures rather than objective indicators. Whilst this could be considered a limitation, we believe that the identification of these subjective reports as predictors has important and positive clinical implications. For a clinician assessing a patient’s risk of anxiety and/or depression in the 12 months after their cardiac event, it is useful to be able to ask the patient simple questions such as these, to identify these risk factors or “red flags” for future mental health problems. A cardiac nurse on the ward, or an allied health professional working in the cardiac rehabilitation setting, will not always have reliable clinical information regarding a previous diagnosis of depression available to them: this information is not systematically included in a medical record. By using a simple question such as “have you been depressed in the past, prior to your cardiac event?”, a clinician is able to easily identify an important red flag to suggest that the patient may be at risk of mental health problems during recovery. Likewise, the use of a simple question to identify financial strain, as used in the present study, could be considered somewhat less intrusive than asking a patient to report their income or their objective financial demands.

Finally, we dichotomized patients’ HADS scores rather than using continuous scale scores to measure anxiety and depression. While the latter could be considered a more statistically powerful approach, it was necessary to dichotomize patients’ HADS scores in order to identify prevalence rates of anxiety and depression over the study period.

### Implications

The present study has identified several risk factors for the development or persistence of anxiety and depression after a cardiac event. By identifying patients early, during hospitalization or at discharge, those at risk can be assisted, potentially mitigating or even preventing future mental health problems. This may in turn reduce the use of health services, therefore reducing costs (Pouwer and Nefs, 2019). Moreover, early identification and treatment has the potential to improve patients’ overall wellbeing and quality of life.

## Data Availability Statement

The datasets generated for this study are available on request to the corresponding author.

## Ethics Statement

The studies involving human participants were reviewed and approved by the Melbourne Health Ethics Committee, Bendigo Health Ethics Committee, and the St John of God Ethics Committee. The patients/participants provided their written informed consent to participate in this study.

## Author Contributions

BM drafted the manuscript. ML, MA, MW, and AJ contributed to the final version. MW and BM designed the study and led the data collection. ML and BM undertook the data analyses. All authors contributed to the interpretation of results and approved the final version of the manuscript.

## Conflict of Interest

The authors declare that the research was conducted in the absence of any commercial or financial relationships that could be construed as a potential conflict of interest.
